# A Retrospective Survey among SARS-CoV-1 Infected Healthcare Workers after Three Years Post-Infection

**DOI:** 10.3390/pathogens10091078

**Published:** 2021-08-25

**Authors:** Szu-Wei Huang, Aspiro Nayim Urbina, Yi-Ming Arthur Chen, Sheng-Fan Wang

**Affiliations:** 1 Model Development Section, Basic Research Laboratory, Center for Cancer Research, National Cancer Institute, Frederick, MD 21702, USA; szu-wei.huang@nih.gov; 2 Center for Tropical Medicine and Infectious Disease, Kaohsiung Medical University, Kaohsiung 80708, Taiwan; aspiro.urbina@hotmail.com; 3 Graduate Institute of Biomedical and Pharmaceutical Science, Fu Jen Catholic University, New Taipei City 242062, Taiwan; 4 Institute of Infectious Diseases and Vaccinology, National Health Research Institutes, Miaoli County 35053, Taiwan; 5 Department of Medical Laboratory Science and Biotechnology, Kaohsiung Medical University, Kaohsiung 80708, Taiwan; 6 Department of Medical Research, Kaohsiung Medical University Hospital, Kaohsiung Medical University, Kaohsiung 80708, Taiwan

**Keywords:** COVID-19, SARS-CoV-1, coronaviruses, convalescent, neutralizing antibodies

## Abstract

Healthcare workers (HCWs) are on the frontline fighting several infectious diseases including SARS-CoV-1 and COVID-19. Coronavirus neutralizing antibodies (nAbs) were recently reported to last for a certain period. The factors affecting nAbs’ existence remain unclear. Here, we retrospectively analyzed the factors correlating with nAbs’ from SARS-CoV-1 long-term convalescence HCWs in Taiwan. One hundred and thirty SARS-CoV-1 convalescent patients were recruited between August 2006 and March 2007. Blood samples were collected to determine the anti-nucleocapsid (N) and anti-spike (S) antibodies’ existence status and neutralization ability. Neutralization ability was measured using SARS-CoV-1 pseudotyped viruses. Statistical analysis of factors associated with anti-SARS-CoV-1 antibodies’ existence status was determined using SAS software. 46.2% SARS-CoV-1 convalescent patients presented anti-N antibody after three years post-infection. Among sixty participants, ten participants co-presented anti-S antibodies. Eight participants with anti-S antibody displayed neutralization ability to SARS-CoV-1. The gender, age, and disease severity of participants did not affect the anti-N antibody existence status, whereas the anti-S antibody is significantly reduced in participants with old age (>50 years, *p* = 0.0434) after three years post SARS-CoV-1 infection. This study suggests that age is an important factor correlated with the duration of SARS-CoV-1 protective antibody existence status.

## 1. Introduction

Healthcare Workers (HCWs) have been on the frontline fighting several infectious diseases, including COVID-19, which may place them at high risk of becoming infected with various emerging or re-emerging diseases. Approximately 20 years ago, Taiwan had experienced a large-scale severe acute respiratory syndrome coronavirus 1 (SARS-CoV-1) outbreak, and many HCWs were infected, resulting in quarantine of HCWs and patients in hospitals, as well as several tertiary hospitals having shut-down [1]. Although many reports have studied SASR-CoV-1 pathogenesis and virus-induced protective neutralizing antibodies (nAbs), the prevalence and influence factors of long-term protective nAbs among SARS-CoV-1 HCWs remain not fully understood.

The SARS-CoV-1 is responsible for the epidemic of severe acute respiratory syndrome (SARS) in 2003. During this time there was a series of outbreaks of SARS-CoV-1 nosocomial infections in Taiwan [1]. According to the World Health Organization, there were 364 SARS-CoV-1 confirmed cases by real-time RT-PCR and/or neutralizing antibodies (nAbs) test in Taiwan in 2003, and among them, 80% were HCWs and patients living in the hospital. When a new pathogen infects humans, it is important to know whether or how long protective immunity can sustain and protect from re-infection. Some studies have reported that the anti-spike (S) antibody in convalescent SARS-CoV-1 infected patients significantly waned after a few years post-infection [2,3]. Furthermore, it is still unclear regarding the deterioration rate of the specific antibodies against SARS-CoV-1, as well as the risk factors (such as age, sex or clinical characteristics) correlated with the anti-SARS antibodies existence status.

Recently, a novel coronavirus (SARS-CoV-2) emerged at the end of 2019 and caused the pandemic of coronavirus infectious disease 2019. The number and level of SARS-CoV-2 infected patients are significantly larger than the SARS-CoV-1. Therefore SARS-CoV-2 is posing a great threat to public health despite several COVID-19 vaccines being applied in different countries [4]. Currently, several data remain lacking, such as the possibility of secondary infection during a short period and how long the protective antibody may sustain. Understanding the duration of immune responses after coronavirus infection could provide valuable information for epidemiological assessments, therapies, and vaccines.

We previously collected the blood samples from SARS-CoV-1 confirmed HCWs after a three-year post-infection and studied the correlation of human-leukocyte antigen typing with SARS-CoV-1 infection [5]. Additionally, a recent report indicated that SARS-CoV-1 and SARS-CoV-2 shared several similarities [6]. The genome of SARS-CoV-2 has shown to be similar to SARS-CoV-1 (79% similarity), with some proteins encoded in SARS-CoV-2 showing higher identity (more than 90%) to SARS-CoV-1 [6]. The nAbs (primarily IgG) binding protein and the spike glycoprotein (S protein) share 77.5% identity between SARS-CoV-2 and SARS-CoV-1 and share 74% identity of the receptor-binding domain. It is believed that the proteins with high identity between SARS-CoV-1 and SARS-CoV-2 have conserved the same mechanisms and functions. The nAbs from convalescent patients infected with SARS-CoV-1 have been reported to be able to cross-neutralize SARS-CoV-2 infection through a blockage of the viral entry step [7,8,9]. This implies that the SARS-CoV-2 and SARS-CoV-1 could present the same protective epitope for producing nAbs. These similarities between SARS-CoV-1 and SARS-CoV-2 draw our interest to address the question regarding the duration of anti-SARS-CoV-1 protective antibodies’ existence status and potential risk factors that may influence antibodies’ expression capabilities. Therefore, we conducted a retrospective survey of nAbs’ existence status using the samples from convalescent SARS-CoV-1 infected HCWs collected after three years post-infection.

## 2. Materials and Methods

### 2.1. Sample Collection

A total of 130 convalescent SARS-CoV-1 infected HCWs were recruited in this study after three years post-infection. The protocols for collecting and processing the blood samples from this cohort were described previously [5,10]. Blood samples of convalescent SARS-CoV-1 infected HCWs were collected from 16 August 2006 to 6 March 2007 (average 3.5 years post-SARS-CoV-1 infection). The serum was separated from blood samples using a centrifuge and stored at −80 °C freezer after the time of collection. 

### 2.2. Neutralization Ability of nAbs

The pseudovirus neutralization assay was conducted according to the protocol published previously [6]. In brief, the SARS-CoV-1 pseudotyped virus normalized for the amount of p24 was incubated with the anti-sera from healthy control group or anti-S or anti-nucleocapsid(N) antibodies from SARS-CoV-1 patients after three years post-infection. After incubation for 1h, these complexes were added to ACE2-transfected 239T cells. The culture was replaced with a fresh medium 24 h later and incubated for an additional 48 h. Luciferase activity was measured according to the manufacturer’s instructions (Promega). The percentage of inhibition assessed by luciferase reporter gene expression was calculated by the reduction in luciferase activity relative to values achieved in the absence of sera. Inhibition seen with control sera was <10% in general compared with samples lacking IgG.

### 2.3. Statistical Analysis

Fisher’s exact and chi-square tests were performed to determine the statistical significance of factors and anti-SARS-CoV-1 antibodies’ existence status. A value of *p* < 0.05 was considered statistically significant. The data analysis was performed using SAS software, Version 9.4 of the SAS System for Windows (SAS Institute, Cary, NC, USA).

## 3. Results

The results indicated that the mean age of participants was 38.7 years (range, 9–81 years). Most of the participants were female (73.1%) and did not present acute respiratory distress syndrome (ARDS) (80%) during SARS-CoV-1 infection (Table 1). To detect the specific antibodies against S or N proteins of SARS-CoV-1, immunoblotting was conducted [1]. The results showed that 46.2% of participants have positive results of anti-N antibody, and 7.7% of participants showed co-existing anti-S and N antibodies after 3 years post SARS-CoV-1 infection (*p* = 0.0003). No anti-S antibodies were detected in participants without anti-N antibodies (Table 1).

**Figure 1:**
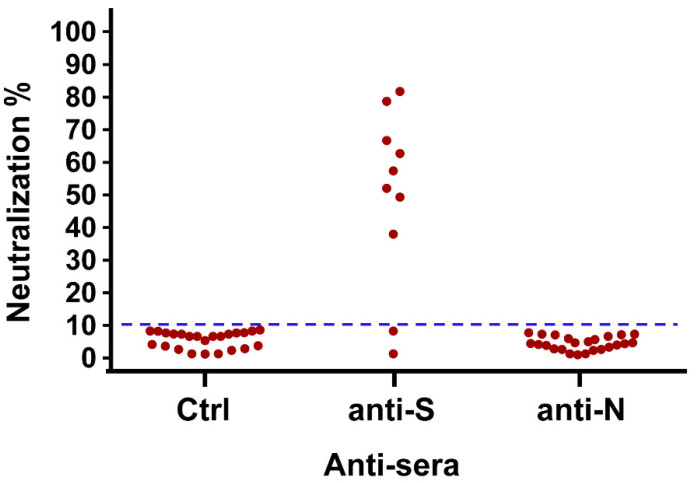
The neutralization ability of anti-sera from convalescent SARS-CoV-1 infected HCWs. The percentage of inhibition assessed by luciferase reporter gene expression was calculated by the reduction in luciferase activity relative to values achieved in the absence of sera. The inhibition seen with control sera was <10% in general compared with samples lacking IgG.

We further performed an antibody neutralization assay using SASR-CoV-1 pseudo-typed virus to determine whether the existing anti-S antibody had the ability to block SARS-CoV-1 infection. Eight anti-S antibodies from participants showed neutralization ability (6.2% of total, 80% of anti-S antibody-positive participants). Due to the limited or loss of serums of the anti-N antibody-positive participants, we only tested 24 participants for neutralization ability. The results indicated that all participants with anti-N antibody did not display neutralizing ability (Figure 1).

Further analysis was done on the factors associated with the existence of anti-SARS-CoV-1 antibodies. Results showed that anti-S antibody was significantly present in younger participants (≤49 years, *p* = 0.043), whereas there was no anti-S antibody in older participants (≥50 years). There are no differences in anti-S antibody existence status with gender or clinical symptom (*p* = 1 and 0.669, respectively) (Table 2). Since the severe disease can provide a higher immune response, we further analyzed the antibodies’ existence status in participants with and without ARDS. However, there are no significant differences in anti-S and anti-N antibodies existence in participants with or without ARDS (*p* = 0.686 and 1, respectively; data not shown).

## 4. Discussion

In this retrospective survey, we found that 46.2% of the participants still had existing anti-N antibodies after three years post SARS-CoV-1 infection. Within the participants with existing anti-N antibodies, there were 16.7% participants, with co-existing anti-S antibody and 80% of them have neutralization ability. Most anti-N antibodies did not show neutralization capabilities; however, a higher percentage of these antibodies (46.2%) were detected in SARS-CoV-1 patients during a long period, suggesting that anti-N antibodies may be good candidates for the measurement of SARS-CoV-1 infection during convalescent phase. Although N protein is not a major target for nAbs generation, it is a representative antigen for the T-cell response to further induce SARS-specific T-cell proliferation and cytotoxic activity [11,12]. The N protein of SARS-CoV-1 and other coronaviruses (such as SARS-CoV-2) are highly conserved and show high immunogenic and abundant expression in infection. The N protein could be a promising target for vaccine development [11,12].

Previous reports indicated that the nAbs from convalescent SARS-CoV-1 infected patients have been shown to start to drop at month 4 and keep decreasing until month 36 post-infection, and it has been further demonstrated that the kinetics of antibodies do not show a significant difference in clinical characteristics [13]. However, to our knowledge, the current study is the first to demonstrate that age is significantly associated with the SARS-CoV-1 nAbs existence status. A small cohort study that recruited 56 convalescent patients with SARS-CoV-1 showed that all participants presented nAbs up to two years post-infection, but the titer decreased significantly after month 16. They found nAbs levels significantly decreased in men compared to women at month 24 after SARS-CoV-1 infection, which suggests that gender may be one of the factors associated with the dynamics of antibody responses [14]. Since the nAbs could significantly wane after two years post-SARS-CoV-1 infection [2], the age difference affecting the nAbs existence status could be observed in convalescent SARS-CoV-1 infected patients after three years post-infection based on our results. Other than the nAbs existence status, the memory B cells and T cells play a vital role against viral re-infection. Several pieces of evidence indicating that the anti-SARS antibodies and peripheral memory B cells responses vanished in convalescent SARS-CoV-1 infected patients after six years post-infection, whereas the SARS-CoV-1 specific memory T cells responses could persist [3,15,16]. Therefore, the SARS-CoV-1 specific memory B cells responses could be a factor affecting the nAbs longevity. The limitations of this study were (1) we do not have multiple points for determining the kinetics of the nAbs and (2) fewer clinical factors and detail medical records of enrolled subjects included in this study. However, there is no significant association linked to the clinical factors and the existence of nAbs after long-term post-SARS-CoV-1 infection reported in other studies.

SARS-CoV-2 pandemic poses great threats for public health and the global economy. Generation of SARS-CoV-2 nAbs from convalescent infected individuals and vaccination is a critical step for prevention of transmission and termination of the pandemic. In convalescent SARS-CoV-2 infected individuals, evidence showed that nAbs can sustain for over a year and exhibited a longer duration of detectable antibody with severe disease patients; however, the nAbs responses were not affected by different age groups [17]. This may be due to individuals infected with SARS-CoV-2 with older age having higher chances to develop severe diseases presentation, thus generating higher titer of nAbs [17,18,19]. We did not observe statistical differences of anti-N or anti-S existence status in HCWs with and without ARDS in our cohort. Similar observation from another study showed that clinical characteristics did not associate with antibody existence status in long-term SARS-CoV-1 convalescent individuals [13]. Currently, two mRNA-based vaccines (Pfizer and Moderna) and two adenoviral vector-based vaccines (Johnson & Johnson and Astra-Zeneca) have been widely applied in several countries [20,21,22,23]. A recent study by Doria-Rose et. al. showed that the mRNA-1273 vaccine-elicited nAbs can sustain at least six months. Intriguingly, they also found nAbs titers were lower in participants with older age (>56 years old) six months after vaccination [24]. SARS-CoV-2 has evolved to several highly transmissible variants, and one of the most contagious variants, the delta variant (B.1.617.2), has become dominant in circulation and can infect fully vaccinated individuals. Evidence showed that individuals receiving two doses of vaccine can provide similar effectiveness against different SARS-CoV-2 variants (included delta variant); however, only one dose vaccination decreased the effectiveness against delta variant [25]. Therefore, it is believed that a booster dose of COVID-19 is needed to fight against upcoming SARS-CoV-2 variants, especially in vulnerable populations.

In conclusion, the current study demonstrated that nAbs significantly decreased in convalescent SARS-CoV-1 infected HCWs after three years post-infection, especially with old age. We, therefore, suggested that continuing to monitor nAbs against SARS-CoV-1 or even the current SARS-CoV-2 existence status in infected or immunized persons should be considered, especially in people with older age.

## Figures and Tables

**Table 1:** Demographic and clinical characteristics of healthcare workers who were convalescent SARS-CoV-1 patients after three years post-infection in this study.

Variable	Anti-N (+) N = 60 *n* (%)	Anti-N (−) N = 70 *n* (%)	*p*
Gender			0.3929 §
Male	14 (23.3)	21 (30.0)	
Female	46 (76.7)	49 (70.0)	
Age			0.1546 *
<20	0 (0.0)	5 (7.1)	
20–29	13 (21.7)	19 (27.1)	
30–39	17 (28.3)	18 (25.7)	
40–49	16 (26.7)	13 (18.6)	
50–59	12 (20.0)	9 (12.9)	
≥60	2 (3.3)	6 (8.6)	
Clinical symptom			1 §
without ARDS	48 (80.0)	56 (80.0)	
ARDS	12 (20.0)	14 (20.0)	
Anti-S Abs			0.0003 *
Negative	50 (83.3)	70 (100.0)	
Positive	10 (16.7)	0 (0.0)	

§, Chi-square test; *, Fisher exact test.

**Table 2:** Factors associated with neutralizing antibody existence status among the positive result of anti-N antibody participants.

Variable	Anti-S (+) N = 10 *n* (%)	Anti-S (−) N = 50 *n* (%)	p *
Gender			1
Male	2 (20.0)	12 (24.0)	
Female	8 (80.0)	38 (76.0)	
Age			0.0434
≤29	1 (10.0)	12 (24.0)	
30–49	9 (90.0)	24 (48.0)	
≥50	0 (0.0)	14 (28.0)	
Clinical symptom			0.6697
without ARDS	9 (90.0)	39 (78.0)	
ARDS	1 (10.0)	11 (22.0)	

* Fisher exact test.

## Data Availability

All data pertaining to the study was described in the manuscript.
